# Empowering Indonesia’s energy transition planning through long-term energy system modelling: A technoeconomic dataset

**DOI:** 10.1016/j.dib.2025.111563

**Published:** 2025-04-15

**Authors:** Mutiara Ramadhani Nur Irbah, Raphael Slade, Mark Howells, Neve Fields, Fernando Antonio Plazas-Niño, Emma Richardson

**Affiliations:** a Centre for Environmental Policy, Imperial College London, Exhibition Road, London SW7 2AZ, United Kingdom; b Centre for Sustainable Transitions: Energy, Environment and Resilience (STEER), Department of Geography, Loughborough University, Epinal Way, Loughborough LE11 3TU, United Kingdom

**Keywords:** Energy planning, Energy modelling, Decarbonisation, OSeMOSYS, FlexTool

## Abstract

As one of the most populous countries globally and a major emerging economy, Indonesia aims to significantly lower its carbon footprint and transform its power sector toward cleaner energy sources. To effectively plan and implement policies to achieve these goals, decision-makers require reliable, comprehensive data on Indonesia's energy systems. However, accessing energy data in Indonesia can be challenging, as is often the case in developing countries where data may be fragmented, out of date, or simply unavailable. In this regard, this data article paper presents an open-access dataset of Indonesia's energy systems to support energy modelling, policy analysis, and research, following the U4RIA goals of Ubuntu, Retrievability, Reusability, Repeatability, Reconstructability, Interoperability, and Auditability. The dataset encompasses historical and projected data from 2010 to 2070 on key aspects such as electricity demand by sector, electricity generation, installed capacity, capacity factors, costs of energy technologies, and renewable energy potentials for solar, wind, hydro, and geothermal power. The data has been collated from Indonesian government reports, international agencies, and academic literature and harmonised using documented assumptions. While Indonesia-specific, this dataset could serve as a template for developing similar energy datasets in other countries, which may face similar challenges in developing energy transition planning.

Specifications Table

**Table:** 

Subject	Engineering & Materials science
Specific subject area	Energy System Modelling
Type of data	Tables and Graphs
Data collection	The data collection process involved a systematic literature review of publicly accessible sources, including scientific journals, government reports, and international agency publications. Key data were extracted from authoritative databases such as the Ministry of Energy and Mineral Resources of Indonesia and complemented by pre-existing modelling databases provided by Climate Compatible Growth. These materials are available on their respective official websites and are fully documented in the references section. Sources were selected based on relevance to Indonesia’s power sector and energy transition, with inclusion criteria covering recency (last 10 years), national relevance, and credibility. Outdated or methodologically unclear datasets were excluded. Analysis was conducted using open-source tools, including OSeMOSYS and FlexTool, to ensure transparency and reproducibility.
Data source location	The data sources are referenced within the relevant sections of this article.
Data accessibility	All the primary data sources are clearly described in the Zenodo repository. Repository name: Zenodo Data identification number: 10.5281/zenodo.14536072 Direct URL to data: https://zenodo.org/records/14536072
Related research article	None

## 1 Value of the Data

• This database consolidates data from multiple sources into a single, harmonised, and easily accessible repository, which could save researchers significant time and effort in data gathering and preprocessing.
• By adhering to the U4RIA principles [1], particularly reconstructability and interoperability, this dataset could allow researchers to easily integrate it with their existing models or utilise it as a foundation for new Indonesia-specific energy models, which may promote further research and collaboration.
• As new energy data becomes available, researchers can reproduce the data processing steps to refine and expand this dataset. This is crucial in the rapidly evolving energy landscape, enabling the research community to maintain a robust, up-to-date resource. Through collaboration, researchers might continuously enhance the quality, scope, and accuracy of publicly available energy data for Indonesia.
• While focused on Indonesia specifically, the dataset could serve as a replicable template for creating similar datasets in other developing countries facing similar energy transition challenges. This may enable cross-country comparisons, knowledge sharing, and the adoption of best practices in energy modelling and analysis.

## 2 Background

Amid growing global momentum toward net-zero emissions, Indonesia has committed to lowering its carbon footprint while transforming its energy systems. To successfully achieve these decarbonisation goals, developing robust evidence-based energy strategies is essential, which fundamentally relies on access to high-quality and comprehensive energy data. However, like many other low- and middle-income countries (LMICs), Indonesia faces significant obstacles in obtaining accessible energy data while also grappling with scattered and fragmented datasets [2]. This data article aims to address these challenges by introducing an open-access dataset on Indonesia’s energy systems that follows U4RIA framework principles (Ubuntu, Retrievability, Repeatability, Reconstructability, Interoperability, and Auditability) [1], thus supporting energy modelling and evidence-based policymaking. This dataset was built to feed into OSeMOSYS and FlexTool models [2,3], so can be readily employed in this context or in similar energy modelling frameworks.

## 3 Data Description

This article introduces a collection of datasets for Indonesia that can be employed in the OSeMOSYS and FlexTool tools to model long-term energy transition planning. It is crucial to understand that these datasets are not tied to these tools and can be utilised independently for various energy research and planning purposes. The datasets have been published publicly and can be retrieved through Zenodo at https://zenodo.org/records/14536072 [3]. The data were compiled from a variety of publicly accessible sources, including reports from the Indonesian government and international agencies, academic publications, and databases of pre-existing models. Specifically, this dataset draws on statistical data from Indonesia’s Ministry of Energy and Mineral Resources, such as Technology Data for the Indonesian Power Sector and Electricity Statistics, as well as the Indonesia Energy Outlook published by the National Energy Council. International sources include the Renewable Energy Outlook for ASEAN by the International Renewable Energy Agency (IRENA). Additional parameters were obtained from peer-reviewed publications and pre-existing techno-economic datasets available through the Climate Compatible Growth (CCG) inventory.

The dataset encompasses a wide range of information, such as electricity demand and generation, capital and fixed costs, operational lifetime, technology efficiencies, capacity factors, residual capacity, renewable energy potential, and key parameters for flexibility modelling for the years 2015 to 2050. These parameters are organised into nine categories, each representing a distinct technology used by Indonesia’s existing power generators. These categories are defined in the “Sets” sheet within the Excel file provided in the repository.

### 3.1 Electricity demand

The electricity demand projections are based on Paiboonsin’s pre-existing model database [4], which expanded upon the “Energy & Transport Starter Data Kits” developed by Climate Compatible Growth (CCG) [5]. As illustrated in Table 1, which shows data for key years only, the electricity demand is disaggregated into three main sectors: commercial, residential, and industrial. Across these sectors, demand is projected to grow at an annual rate of 2–5 % [4]. To access the full demand dataset for complete timeseries data if needed, users can refer to the "Demand" sheet within the Excel file available in the repository.

**Table 1:** Total electricity demand per sector for key years (PJ).

Year	Industrial	Residential	Commercial	Total Electricity Demand
*2015*	261.17	323.97	192.77	777.91
*2020*	337.55	418.71	249.15	1005.42
*2025*	405.16	502.58	299.06	1206.80
*2030*	512.09	635.22	377.98	1525.29
*2035*	671.76	833.28	495.84	2000.87
*2040*	834.24	1034.82	615.76	2484.82
*2045*	991.25	1229.59	731.66	2952.49
*2050*	1117.80	1386.57	825.06	3329.43

### 3.2 Costs

The primary sources for capital and fixed cost data are the “Technology Data for the Indonesian Power Sector: Catalogue for Generation and Storage of Electricity” annual report by the Ministry of Energy and Mineral Resources (MEMR) of Indonesia [6] and the “National Hydrogen Strategy” published by MEMR [7]. This report covers data for various technologies, including all hydropower types, SCGT, CCGT, light fuel oil, biomass, offshore and onshore wind, geothermal, solar PV (utility), and coal for the years 2020, 2030, and 2050. Data for additional technologies not covered in the report is derived from the pre-existing model dataset [4]. The entire dataset can be accessed in the “Capital Cost” and “Fixed Cost” sheets of the Excel file in the repository (Table 2, Table 3).

**Table 2:** Capital costs of key years ($/kW/year).

Technology	2015	2020	2025	2030	2035	2040	2045	2050
Biomass Power Plant	2280	2280	2220	2070	2007.5	1945	1882.5	1820
Coal Power Plant	1736.67	1736.67	1721.42	1683.3	1669.98	1656.65	1643.33	1630
Geothermal Power Plant	5500	5500	5500	5500	5362.5	5225	5087.5	4950
Light Fuel Oil Power Plant	910	910	910	910	905	900	895	890
Oil Fired Gas Turbine (SCGT)	1344	1344	1344	1344	1344	1344	1344	1344
Gas Power Plant (CCGT)	1080	1080	1065.71	1030	1010	990	970	950
Gas Power Plant (SCGT)	1120	1120	1102.86	1060	1042.5	1025	1007.5	990
Solar PV (Utility)	960	960	877.143	670	622.5	575	527.5	480
CSP with Storage	7404.71	4965.31	4000	3223.13	3178.59	3134.9	3134.9	3134.9
Large Hydropower Plant (Dam) (>100MW)	2200	2200	2174.29	2110	2072.5	2035	1997.5	1960
Medium Hydropower Plant (Dam) (10-100MW)	2500	2500	2471.43	2400	2357.5	2315	2272.5	2230
Small Hydropower Plant (Dam) (<10MW)	2700	2700	2668.57	2590	2542.5	2495	2447.5	2400
Onshore Wind	1650	1650	1521.43	1200	1137.5	1075	1012.5	950
Offshore Wind	4100	4100	3948.57	3570	3395	3220	3045	2870
Nuclear Power Plant	5500	5500	5500	5500	5500	5500	5500	5500
Utility-scale PV with 2 hour storage	2785.5	1869	1287.33	1079.83	975.44	877.622	844.622	812.622
Onshore Wind power plant with storage	3294.52	2466.36	1935.13	1736.61	1496.17	1484.71	1472.84	1462.43
Light Fuel Oil Standalone Generator (1 kW)	1500	1500	1500	1500	1500	1500	1500	1500
Solar PV (Distributed with Storage)	3502	2130.8	1880.8	1755.8	1723.8	1690.8	1657.8	1625.8
Off-grid Hydropower	2162	2162	2162	2162	2162	2162	2162	2162
PEM Electrolyser	1430	1430	1282.33	1100	975	850	725	600
PEM Fuel Cell	1670	1670	1318.55	880	797.5	715	632.5	550
Pumped Hydro Storage	1200	1200	1200	1200	1200	1200	1200	1200
Electricity Transmission	204.267	204.267	204.267	204.267	204.267	204.267	204.267	204.267
Electricity Distribution	102.133	102.133	102.133	102.133	102.133	102.133	102.133	102.133

**Table 3:** Fixed costs of key years ($/kW/year).

Technology	2015	2020	2025	2030	2035	2040	2045	2050
Biomass Power Plant	54	54	52.7714	49.7	48.075	46.45	44.825	43.2
Coal Power Plant	54.37	54.37	53.8929	52.7	52.295	51.89	51.485	51.08
Geothermal Power Plant	145	145	145	145	141.375	137.75	134.125	130.5
Light Fuel Oil Power Plant	9.12	9.12	9.12	9.12	9.05175	8.9835	8.91525	8.847
Oil Fired Gas Turbine (SCGT)	18	18	18	18	18	18	18	18
Gas Power Plant (CCGT)	26.8	26.8	26.5714	26	25.8	25.6	25.4	25.2
Gas Power Plant (SCGT)	26.5	26.5	26.2714	25.7	25.5	25.3	25.1	24.9
Solar PV (Utility)	7.5	7.5	7.3	6.8	6.625	6.45	6.275	6.1
CSP with Storage	120	120	120	120	120	120	120	120
Large Hydropower Plant (Dam) (>100MW)	43	43	42.4286	41	40.25	39.5	38.75	38
Medium Hydropower Plant (Dam) (10-100MW)	47.8	47.8	47.2286	45.8	44.975	44.15	43.325	42.5
Small Hydropower Plant (Dam) (<10MW)	60.4	60.4	59.7143	58	57	56	55	54
Onshore Wind	40	40	38.8571	36	34.5	33	31.5	30
Offshore Wind	118.8	118.8	113.371	99.8	95.175	90.55	85.925	81.3
Nuclear Power Plant	138	138	138	138	138	138	138	138
Utility-scale PV with 2 hour storage	27.855	18.69	12.8733	10.7983	9.7544	8.77622	8.44622	8.12622
Onshore Wind power plant with storage	131.781	98.6544	77.4052	69.4644	59.8468	59.3884	58.9137	58.497
Light Fuel Oil Standalone Generator (1 kW)	38	38	38	38	38	38	38	38
Solar PV (Distributed with Storage)	70.04	42.616	37.616	35.116	34.476	33.816	33.156	32.516
Off-grid Hydropower	64.86	64.86	64.86	64.86	64.86	64.86	64.86	64.86
PEM Electrolyser	40	40	40	40	40	40	40	40
PEM Fuel Cell	20	20	20	20	20	20	20	20
Pumped Hydro Storage	18.7	18.7	18.7	18.7	18.7	18.7	18.7	18.7
Electricity Transmission	4.08533	4.08533	4.08533	4.08533	4.08533	4.08533	4.08533	4.08533
Electricity Distribution	2.04267	2.04267	2.04267	2.04267	2.04267	2.04267	2.04267	2.04267

### 3.3 Operational lifetime

The operational lifetime data, which indicates the typical operational lifespan of a power plant, is also sourced from the MEMR’s annual report [6], covering multiple technologies, including all types of hydropower, SCGT, CCGT, light fuel oil, biomass, offshore and onshore wind, geothermal, solar PV (utility), and coal. For the remaining technologies, data was sourced from an earlier model dataset [4]. The data is provided in the “Operational Life” sheet of the repository’s Excel file and presented in Table 4.

**Table 4:** Operational lifetime (years).

Technology	Operational Lifetime (years)
Biomass Power Plant	25
Coal Power Plant	30
Geothermal Power Plant	30
Light Fuel Oil Power Plant	25
Oil Fired Gas Turbine (SCGT)	50
Gas Power Plant (CCGT)	25
Gas Power Plant (SCGT)	25
Solar PV (Utility)	35
CSP with Storage	35
Large Hydropower Plant (Dam) (>100 MW)	50
Medium Hydropower Plant (Dam) (10–100 MW)	50
Small Hydropower Plant (Dam) (<10 MW)	50
Onshore Wind	27
Offshore Wind	27
Nuclear Power Plant	60
Utility-scale PV with 2 h storage	30
Onshore Wind power plant with storage	30
Light Fuel Oil Standalone Generator (1 kW)	20
Solar PV (Distributed with Storage)	41
Off-grid Hydropower	40
PEM Electrolyser	20
PEM Fuel Cell	20
Pumped Hydro Storage	60
Electricity Transmission	50
Electricity Distribution	70

### 3.4 Technology efficiencies

Technology efficiency refers to the ratio of useful energy output to the total energy input for a specific technology or process. It measures how effectively a particular technology converts input energy (such as fuel) into the desired output energy (such as electricity). The study used technology efficiency from the pre-existing model database [4] that compiles the data published by the ASEAN Centre for Energy with IRENA [8], with base-year estimates primarily from 2015. These efficiency values are applied uniformly across the entire modelling period from 2015 to 2070, with no projections or learning rates incorporated due to uncertainty regarding future technological advancements specifically in the Indonesian context. This approach ensures transparency and avoids introducing speculative assumptions into the model. The data is provided in the “Technology Efficiency” sheet of the repository’s Excel file and presented in Table 5.

**Table 5:** Technology efficiencies.

Technology	Efficiency (%)
Biomass Power Plant	38.02
Coal Power Plant	30.03
Geothermal Power Plant	10.00
Light Fuel Oil Power Plant	40.00
Oil Fired Gas Turbine (SCGT)	40.00
Gas Power Plant (CCGT)	55.25
Gas Power Plant (SCGT)	34.60
Solar PV (Utility)	100.00
CSP with Storage	100.00
Large Hydropower Plant (Dam) (>100MW)	100.00
Medium Hydropower Plant (Dam) (10-100MW)	100.00
Small Hydropower Plant (Dam) (<10MW)	100.00
Onshore Wind	100.00
Offshore Wind	100.00
Nuclear Power Plant	33.00
Utility-scale PV with 2 hour storage	100.00
Onshore Wind power plant with storage	100.00
Light Fuel Oil Standalone Generator (1 kW)	42.02
Solar PV (Distributed with Storage)	100.00
Off-grid Hydropower	100.00

### 3.5 Emission factors

**Fig. 1:**
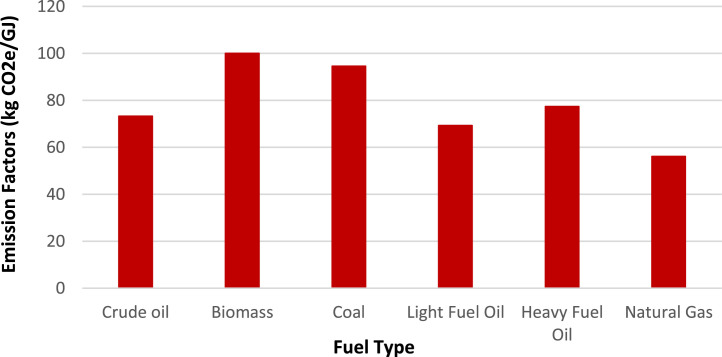
Emission factors by fuel type.

The emission factor measures how much pollution a technology produces relative to its activity level, showing emissions released per unit of operation. Throughout their operational lifespan, power plants release various greenhouse gases, including nitrous oxides, methane, and carbon dioxide. For this research, these emissions are specifically measured in carbon dioxide equivalent (CO_2_e). This study used emission factors from a pre-existing model database [4] that compiles emission data documented by the IPCC [9]. The data is provided in the “CO2 Emission” sheet of the repository’s Excel file and presented in Table 6 (Fig. 1).

**Table 6:** Emission factors.

Technology	Fuel	CO_2_ Emission Factors (kg CO_2_e/GJ)
Crude Oil Imports	Crude oil	73.3
Crude Oil Extraction	Crude oil	73.3
Biomass Imports	Biomass	100
Biomass Extraction	Biomass	100
Coal Imports	Coal	94.6
Coal Extraction	Coal	94.6
Light Fuel Oil Imports	Light Fuel Oil	69.3
Heavy Fuel Oil Imports	Heavy Fuel Oil	77.4
Natural Gas Imports	Natural Gas	56.1
Natural Gas Extraction	Natural Gas	56.1

### 3.6 Capacity factor

The capacity factor indicates how effectively a power generator is utilised by comparing its actual energy production over time against its maximum potential output. Table 7 provides an overview of the capacity factor data for various power generation technologies across different timeslices, which can also be found in the “Capacity Factor & Demand” sheet of the Excel file stored in the repository. The timeslices represent specific temporal divisions used in the energy model, based on Indonesia's equatorial climate patterns: S101 represents the daytime during the dry season (November–March, 6 AM-6 PM), S102 represents the nighttime during the dry season (November–March, 6 PM-6 AM), S103 represents the daytime during the wet season (April–October, 6 AM-6 PM), and S104 represents the nighttime during the wet season (April–October, 6 PM-6 AM). This data is calculated based on assumptions and methods that are obtained from the previous model database [4].

**Table 7:** Capacity factor.

Technology	Timeslices	Capacity Factor
Biomass Power Plant	S101–S104	0.7
Coal Power Plant	S101–S104	0.75
Geothermal Power Plant	S101–S104	0.7
Light Fuel Oil Power Plant	S101–S104	0.25
Oil Fired Gas Turbine (SCGT)	S101–S104	0.25
Gas Power Plant (CCGT)	S101–S104	0.55
Gas Power Plant (SCGT)	S101–S104	0.55
Solar PV (Utility)	S101	0
	S102	0.369931481
	S103	0
	S104	0.372241848
CSP with Storage	S101	0.05
	S102	0.3
	S103	0.05
	S104	0.3
Large Hydropower Plant (Dam) (>100MW)	S101–S102	0.551496241
	S103–S104	0.42004426
Medium Hydropower Plant (Dam) (10-100MW)	S101–S102	0.551496241
	S103–S104	0.42004426
Small Hydropower Plant (Dam) (<10MW)	S101–S102	0.551496241
	S103–S104	0.42004426
Onshore Wind	S101	0.034587963
	S102	0.030465741
	S103	0.030161232
	S104	0.019900362
Offshore Wind	S101–S104	0.2
Nuclear Power Plant	S101–S104	0.825
Utility-scale PV with 2 hour storage	S101	0.061655247
	S102	0.369931481
	S103	0.062040308
	S104	0.372241848
Onshore Wind power plant with storage	S101–S104	0.034948126
Light Fuel Oil Standalone Generator (1 kW)	S101–S104	0.4
Solar PV (Distributed with Storage)	S101	0.061655247
	S102	0.369931481
	S103	0.062040308
	S104	0.372241848
Off-grid Hydropower	S101–S102	0.551496241
	S103–S104	0.42004426

### 3.7 Residual capacity

The residual capacity parameter indicates the total installed capacity that existed before the modelling timeframe begins, representing all infrastructure and equipment already in place at the starting point of the analysis. Data on existing on-grid capacity is drawn primarily from the Global Energy Monitor’s database of Indonesian power facilities [10]. This raw data is then calculated to derive the final values, which will be described later in Section 3.5. Moreover, for off-grid technologies and the capacity of electricity transmission and distribution networks, the data is sourced from a previous model dataset [4]. The calculated residual capacity values are summarised in Table 8, with detailed data provided in the “Residual Capacity” sheet of the repository’s Excel file.

**Table 8:** Residual capacity of key years (GW).

Technology	2015	2020	2025	2030	2035	2040	2045	2050
Biomass Power Plant	1.74	1.762	1.74	1.74	0	0	0	0
Coal Power Plant	24.5966	35.6026	48.5916	43.3466	43.3166	38.145	26.184	14.648
Geothermal Power Plant	1.351	1.942	1.96	1.525	1.505	1.167	0.939	0.238
Light Fuel Oil Power Plant	6.275	4.864	4.987	4.987	4.987	4.987	4.987	4.987
Oil Fired Gas Turbine (SCGT)	0.819	3.178	3.207	3.207	3.207	3.207	3.207	3.207
Gas Power Plant (CCGT)	10.146	12.236	13.799	13.799	13.799	13.799	13.799	13.799
Gas Power Plant (SCGT)	4.311	5.348	5.348	5.348	5.348	5.348	5.348	5.348
Solar PV (Utility)	0.009	0.1473	0.2011	0.2011	0.2011	0.2011	0.2011	0.2011
CSP with Storage	0	0	0	0	0	0	0	0
Large Hydropower Plant (Dam) (>100MW)	5.079	5.638	4.53941	4.53941	4.53941	4.53941	4.53941	4.53941
Medium Hydropower Plant (Dam) (10-100MW)	0.151	0.413	0.505	0.505	0.505	0.505	0.505	0.505
Small Hydropower Plant (Dam) (<10MW)	0.03	0.106	0.042	0.042	0.042	0.042	0.042	0.042
Onshore Wind	0.00112	0.15431	0.01	0.01	0.01	0	0	0
Offshore Wind	0	0	0	0	0	0	0	0
Nuclear Power Plant	0	0	0	0	0	0	0	0
Utility-scale PV with 2 hour storage	0	0	0	0	0	0	0	0
Onshore Wind power plant with storage	0	0	0	0	0	0	0	0
Light Fuel Oil Standalone Generator (1 kW)	0	0	0	0	0	0	0	0
Solar PV (Distributed with Storage)	0.04162	0.04508	0.04447	0.04287	0.02926	0.00337	0.00207	0.00207
Off-grid Hydropower	0.01442	0.01485	0.01485	0.01485	0.01485	0.01203	0.01025	0.00771
PEM Electrolyser	0	0	0.5	0.4	0	0	0	0
PEM Fuel Cell	0	0	0.5	0.4	0	0	0	0
Pumped Hydro Storage	0	0	0	0.76	0	0	0	0
Electricity Transmission	58.9	58.9	58.9	58.9	58.9	58.9	58.9	58.9
Electricity Distribution	58.9	58.9	58.9	58.9	58.9	58.9	58.9	58.9

### 3.8 Electricity production

**Fig. 2:**
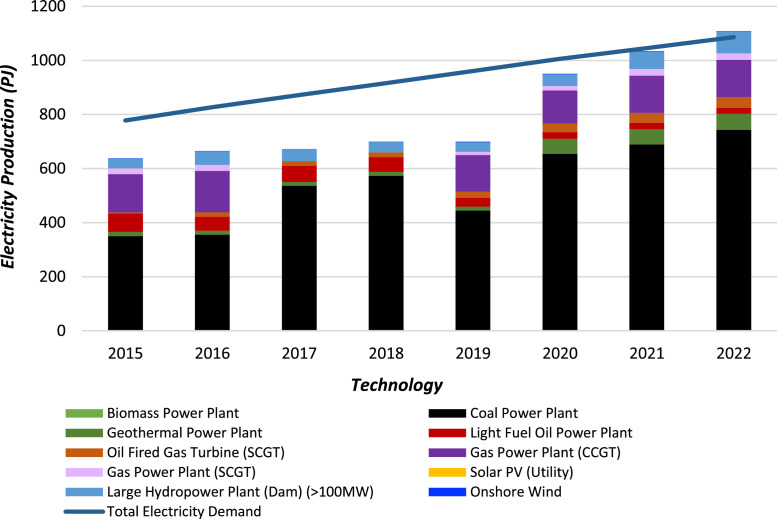
Electricity production by technology in Indonesia (2015–2022).

Power generation data for each technology between 2015 and 2022 is obtained from MEMR’s annual report [11]. The dataset covers electricity production at the national level, encompassing major interconnected grids and large standalone systems across Indonesia. The figures include utility-scale generation from power plants operated by PLN, the state-owned electricity company, as well as independent power producers. The data is presented in Table 9 and stored in the “Electricity Production” sheet of the repository’s Excel file (Fig. 2).

**Table 9:** Electricity production for each technology (PJ).

Technology	2015	2016	2017	2018	2019	2020	2021	2022
Biomass Power Plant	1.6596	2.1024	1.9224	1.8936	0.7884	0.702	0.7992	0.5724
Coal Power Plant	348.32	353.74	534.11	571.59	444.15	653.89	688.08	742.334
Geothermal Power Plant	15.81	14.25	14.75	14.45	14.8	56.03	57.23	60.0372
Light Fuel Oil Power Plant	67.89	51.61	59.52	54.63	32.59	24.23	23.12	21.888
Oil Fired Gas Turbine (SCGT)	4.44	17.23	17.23	17.23	22.15	32.69	37.27	38.9016
Gas Power Plant (CCGT)	141.54	152.56	0	0	135.93	121.22	136.71	138.51
Gas Power Plant (SCGT)	21.27	22.31	0	0	11.57	17.11	25.15	23.6052
Solar PV (Utility)	0.02	0.02	0.02	0.02	0.02	0.02	0.43	0.78383
CSP with Storage	0	0	0	0	0	0	0	0
Large Hydropower Plant (Dam) (>100MW)	36.02	49.99	44.73	38.62	35.56	43.02	63.9	80.478
Onshore Wind	0.0144	0.0216	0	0.6768	1.7352	1.7028	1.566	1.2744

### 3.9 Renewable energy potential

Indonesia possesses various renewable energy sources, including solar, wind, geothermal, hydropower, and bioenergy. Data regarding both the potential and actual utilisation of these renewable resources comes from the “Indonesia Energy Outlook” annual report published by Indonesia’s National Energy Council [12]. This information is presented in Table 10 and can also be found in the “RE Supply & Potential” sheet within the repository’s Excel file.

**Table 10:** Renewable energy potential.

Renewable Energy	Potential (GW)	Realisation / Power Plant Capacity (GW)	Realisation (%)
Tidal	60.0	0	0
Geothermal	23.0	2.36	10.26 %
Bioenergy	57.0	3.13	5.49 %
Wind	155.0	0.15	0.10 %
Hydropower	95	6.69	7.04 %
Solar	3,295	0.31	0.01 %

**Total**	**3,685.0**	**12.64**	**0.34** %

### 3.10 Parameters for flexibility modelling

The data input for FlexTool is primarily organised across two key sheets: “master” and “unit_type”. The “master” sheet contains information on core parameters and settings that impact the entire model. Meanwhile, the “unit_type” sheet contains the parameters specifying all the technologies that make up the system. As this study focuses solely on the electricity grid, all the technologies considered are electricity generation units. The data used in this study is sourced from publicly accessible reports and databases [13,14]. When Indonesia-specific data is unavailable for certain inputs, the data is obtained from published academic literature [[13], [14], [15], [16], [17], [18]]. The values for the “master” sheet and “unit_type” sheet are summarised in Table 11, Table 12, respectively, and can be found in the “Key Parameters FlexTool” sheet within the repository’s Excel file.

**Table 11:** Key parameters for the “master” sheet in FlexTool.

Parameter	Value
CO_2_ Cost (USD/Tonne)	2
Loss of Load Penalty (USD/MWh)	3970
Loss of Reserves Penalty (USD/MWh)	1000
Lack of Inertia Penalty (USD/MWh)	30,000
Curtailment Penalty (USD/MWh)	11
Lack of Capacity Penalty (USD/MWh)	5000
Time Modelled (Year)	1

**Table 12:** Key parameters for the “unit_type” sheet in FlexTool.

Technology	Min. Load (%)	Ramp Up (p.u. per min)	Ramp Down (p.u. per min)	Inertia Constant (MWs/MW)	Min. Uptime (hour)	Min. Downtime (hour)
Biomass Power Plant	0.252	0.013	0.013	32	2	2
Coal Power Plant	0.43	0.074	0.074	2.632	34	34
Gas Power Plant	0.24	0.084	0.084	4.692	24	24
Geothermal Power Plant	0.413	0.033	0.033	1.063	13	13
Oil Fired Gas Turbine (SCGT)	0.23	0.553	0.553	6.23	13	13
Solar PV (Utility)	*NA	13	13	3	*NA	*NA
Hydropower Plant	0.054	0.153	0.153	2.793	13	13
Onshore Wind	*NA	13	13	3	*NA	*NA

*NA = not applicable

## 4 Experimental Design, Materials and Methods

This section details the sources and processing methods used for specific techno-economic parameters within the dataset. Building upon a pre-existing model dataset, the information has been updated using the most recent data from a wide range of sources. These include databases and reports from international organizations such as the International Renewable Energy Agency (IRENA), and national energy agencies such as Indonesia’s Ministry of Energy and Mineral Resources, through publications like Electricity Statistics and Technology Data for the Indonesian Power Sector, as well as the National Energy Council, through the Indonesia Energy Outlook. Academic sources include peer-reviewed literature on capacity expansion, energy storage, and grid flexibility modelling, published in journals such as Energies, Journal of Energy Storage, and Energy Strategy Reviews. Once collected, the raw data underwent a systematic process of organisation, analysis, and standardisation to meet modelling specifications.

### 4.1 Electricity demand and capacity factor

The raw electricity demand data is sourced from the pre-existing model dataset [4], while the capacity factor data is calculated by reducing the number of time slices from the original eight time slices to four. This reduction reflects Indonesia’s equatorial climate patterns: the dry season (November–March) and wet season (April–October), combined with daily cycles (daytime: 6 AM-6 PM and nighttime: 6 PM–6 AM). This resulted in four distinct time slices: Dry-Day (S101), Dry-Night (S102), Wet-Day (S103), and Wet-Night (S104). The methodology for reducing these time slices followed Cannone et al.’s approach [19].

### 4.2 Costs

The primary source for capital and fixed costs data in this study is the “Technology Data for the Indonesian Power Sector: Catalogue for Generation and Storage of Electricity” annual report by MEMR [6]. While this source document contains specific figures until 2022 and forecasts for 2030 and 2050, this study filled in the gaps for other years using the linear projections formula in Excel. These projections span from 2023 to 2070 and were calculated using a constant rate derived from the 2030 and 2050 forecasted values.

### 4.3 Electricity production and technology efficiency

The electricity production and technology efficiency data are not utilised in their raw form. Historical electricity production figures up through 2022 were taken from MEMR’s “Electricity Statistics of Indonesia” annual report [11], which provided the data in Gigawatt-hours (GWh). These values were then converted to Petajoules (PJ) to ensure compatibility with the unit used in the OSeMOSYS model.

Meanwhile, although technology efficiency is not utilised as a direct input for OSeMOSYS, it is used to calculate the “Input Activity Ratio” parameter, which the model does require. This ratio determines the input units needed per unit of activity for a given technology. For instance, if a natural gas power plant operates at 50 % efficiency and produces one unit of electricity, it requires two units of natural gas input (calculated as 1/50 %). Therefore, in this scenario, the Input Activity Ratio parameter in OSeMOSYS would be set to 2, representing the relationship between the natural gas input and electricity output.

### 4.4 Operational lifetime, renewable energy potential, and emission factors

Unlike some other parameters, some key sets were used in their original form, including operational lifetime, renewable energy potential, and emission factors. As described in Section 2, these values came from various sources and were input directly into the OSeMOSYS model. The only change made in this study is updating the data based on the most recent annual reports published by the Indonesian government.

### 4.5 Residual capacity

For off-grid technologies, the residual capacity data is obtained from a pre-existing model dataset, and the raw data is used directly as the model input without any adjustments. On the other hand, the primary source for data on existing on-grid technologies is the Global Energy Monitor’s database [10]. This database provides information about regional power plants in Indonesia, such as their installed capacity, commissioning dates, and projected retirement dates. The database includes both power plants that are currently in operation and those that have been decommissioned since the base year of the model.

Once the data has been collected, it undergoes data processing using the Residual Capacity Calculator workbook, which can be accessed through Zenodo via this link: https://zenodo.org/records/11475824 [20]. To determine the residual capacity for a given year and technology, the capacities of all currently operating power plants of that technology type are added together. The retirement timeline for each plant is then taken into account. Once a plant reaches its assumed retirement year, its capacity is excluded from the residual capacity calculation for that year and all subsequent years.

## Limitations

This dataset has limitations, including limited data on off-grid electricity generation and power plant capacity, which results in a primary focus on accessible on-grid data with few off-grid inclusions. The reliance on open-source data requires assumptions, and generic flexibility parameters could potentially limit the model’s precision. Including other off-grid technologies and enhancing data accuracy would be beneficial for developing more robust energy modelling.

## Ethics Statement

The authors of this dataset have adhered to the ethical standards for publication in Data in Brief. They affirm that the study does not include participation of human subjects, animal experiments, or data from social media platforms.

## CRediT Author Statement

**Mutiara Ramadhani Nur Irbah**: Conceptualization, Methodology, Formal analysis, Investigation, Data curation, Writing – Original draft preparation. **Raphael Slade**: Supervision. **Mark Howells**: Supervision. **Neve Fields**: Supervision, Writing – Review & editing. **Fernando Antonio Plazas-Niño**: Project administration, Writing – Review & editing. **Emma Richardson**: Project administration.

## U4RIA Compliance Statement

This work follows the U4RIA guidelines which provide a set of high-level goals relating to conducting energy system analyses in countries [1]. This paper was carried out involving stakeholders in the development of models, assumptions, scenarios and results (Ubuntu / Community). The authors ensure that all data, source code and results can be easily found, accessed, downloaded, and viewed (retrievability), licensed for reuse (reusability), and that the modelling process can be repeated in an automatic way (repeatability). The authors provide complete metadata for reconstructing the modelling process (reconstructability), ensuring the transfer of data, assumptions and results to other projects, analyses, and models (interoperability), and facilitating peer-review through transparency (auditability).

## Data Availability

ZenodoTechnoeconomic Data and Assumptions for Energy Systems Modelling (OSeMOSYS and FlexTool) in Indonesia (Original data)
